# Comparative clinical and radiographic outcomes between early and delayed wrist mobilization after volar fixed-angle plate fixation of distal radius fracture

**DOI:** 10.1038/s41598-022-13909-4

**Published:** 2022-06-10

**Authors:** Panai Laohaprasitiporn, Kitidate Boonchai, Yuwarat Monteerarat, Roongsak Limthongthang, Torpon Vathana

**Affiliations:** Department of Orthopaedic Surgery, Faculty of Medicine Siriraj Hospital, Mahidol University, Bangkok, Thailand

**Keywords:** Orthopaedics, Randomized controlled trials

## Abstract

Postoperative immobilization protocols after volar fixed-angle plate fixation of distal radius fractures (DRF) vary among surgeons. This study aimed to compare functional outcomes, radiographic parameters, and complications between early and delayed mobilization after volar fixed-angle plate fixation of DRF. This study is a randomized controlled trial. The early group was allowed to perform wrist motion exercise immediately after surgery and the delayed group was allowed to perform it after 2 weeks of external immobilization. Postoperative patient-rated wrist evaluation (PRWE), disabilities of arm, shoulder, and hand (DASH), wrist range of motion, visual analog scale (VAS) pain score, and grip strength were evaluated. Forty-eight patients with DRF were enrolled and randomly allocated to the early or delayed mobilization groups. The PRWE, DASH, VAS pain score, grip strength, and wrist motion of both groups significantly improved over time. However, there were no significant differences between groups at any timepoint. Radiographic parameters were not different between groups. There were no significant differences in functional outcomes, radiographic parameters, and complications between early and delayed mobilization after volar fixed-angle plate of DRF. Immediate postoperative wrist range-of-motion exercise can be safely initiated after volar fixed-angle plate fixation of DRF without external immobilization.

**Clinical trial registration:** Thaiclinicaltrials.org identifier: TCTR20180927005. Registered 27/09/2018—retrospectively registered. https://www.thaiclinicaltrials.org/show/TCTR20180927005.

## Introduction

Distal radius fracture (DRF) is one of the most common fractures of upper extremities in adult patients. Its incidence is 17.5% of all fractures in adults, and it is abruptly increased in the elderly with osteoporosis^1^. Volar fixed-angle plate fixation has gained popularity as a surgical option for DRF and functional outcomes in the early phase are better compare to those of other surgical procedures^2^. The advantages of volar fixed-angle plate include the possibility of realigning the displaced fracture to an anatomical position and that of creating a stable fracture fixation. Many biomechanical studies have proven the stability of volar fixed-angle plate fixation^3–5^. Theoretically, the implant is stable enough to initiate an early rehabilitation protocol and an early return to daily activity^6–8^. However, postoperative immobilization protocols are varied among surgeons, ranging from no external immobilization to the application of a short arm slab or cast for 2–7 weeks^9,10^. Some surgeons prefer postoperative immobilization to prevent fracture displacement and decrease pain after the operation, while others prefer early mobilization to prevent joint stiffness and promote an early return to daily activities.

According to a variety of rehabilitation protocols after DRF fixation, results might vary among studies. Therefore, this study aimed to compare functional outcomes and radiographic parameters between early and delayed mobilization protocols after volar fixed-angle plate fixation of DRF. The primary outcome of this study was the patient-rated wrist evaluation (PRWE) score 3 months after the index operation. Additionally, disabilities of the arm, shoulder, and hand (DASH) score, visual analog scale (VAS) pain score, wrist range of motion, grip strength, radiographic parameters, and complications were also evaluated.

## Materials and methods

The study was a parallel design, randomized controlled trials conducted at Faculty of Medicine Siriraj Hospital, Mahidol University, Bangkok, Thailand between June 2017 and May 2019. The study protocol was approved by the institutional review board of research involving human subjects. Written informed consent was obtained from all included participants. This study was performed in accordance with the Declaration of Helsinki and reported according to Consolidated Standards of Reporting Trials (CONSORT).

All patients with DRF were screened for enrollment according to inclusion and exclusion criteria. The inclusion criteria were patients with DRF over 18 years old who received volar fixed-angle plate fixation within 4 weeks after injury. The exclusion criteria were unstable DRF after volar fixed-angle plate fixation identified by intraoperative fluoroscopic examination and needing further postoperative immobilization. We excluded patients with ulnar styloid fracture or the presence of distal radio-ulnar joint instability needing additional ulnar styloid fixation or triangular fibrocartilage complex repair. Patients with pathologic fractures, open fractures, previous wrist fractures, and multiple traumas were excluded from the study. Severe cognitive impairment patients which could not comply with postoperative rehabilitation programs or whose condition might affect the outcome assessments were also excluded.

Participants were consecutively enrolled and randomized into one of the two groups (early and delayed mobilization groups) by a block-of-4 randomization sequence. The randomization sequences were concealed by using sequentially numbered, opaque sealed envelopes. The envelopes were opened right after surgery by registered nurse. All participants were operated with the same operative technique using a flexor carpi radialis approach. After adequate fixation, wrist motions in flexion, extension, ulnar deviation, and radial deviation were assessed under dynamic fluoroscopic examination to ensure the stability of the fracture fixation.

### Postoperative rehabilitation protocols and medications

The early group was instructed to perform postoperative wrist flexion-extension and pronation-supination motion exercises as soon as possible under loose sterile dressing without external immobilization. The delayed group was immobilized with a short arm volar slab for two weeks after surgery. All participants were allowed to do range-of-motion exercises of the finger, elbow, and shoulder joints. Participants demonstrated the instructed wrist motion exercise to the instructors to ensure the correct method of rehabilitation. Stitches were removed at 2 weeks after surgery. At the 2-week postoperative follow-up, short arm volar slabs of the delayed group were removed, and participants were instructed to perform wrist flexion-extension and pronation-supination motion exercises. The same postoperative pain control medications were prescribed in all participants, which included 250 mg of naproxen orally twice a day for three days and 500 mg of paracetamol orally as needed. If the patients had a contraindication to nonsteroidal anti-inflammatory drugs, 50 mg of tramadol orally three times a day for three days were prescribed instead of naproxen.

### Outcome assessments

Outcome assessments in the study were divided into three types: patient-reported outcomes, clinical assessments, and radiographic assessments. Patient-reported outcomes comprised PRWE and DASH questionnaires. Clinical assessments were wrist range of motion, grip strength, and VAS pain score. The clinical outcomes were blindly assessed by research assistant. Radial inclination, ulnar variance, and radial tilt were measured from an anteroposterior view and a lateral view of wrist plain radiograph. The primary outcome of this study was the PRWE score at the 3-month follow-up. The PRWE was measured at 2 weeks, 6 weeks, 3 months, and 12 months. The other outcome measurements were assessed at 2 weeks, 6 weeks, and 3 months after surgery. Postoperative complications (i.e., surgical site infection, carpal tunnel syndrome, wound dehiscence, skin blebs, and complex regional pain syndrome) were assessed at every follow-up visit.

### Patient-rated wrist evaluation (PRWE)

PRWE is a self-reported assessment of pain at wrist joint and functional difficulties in daily activities. The psychometric properties of the PRWE questionnaire have been studied in patients with DRF and reported to have good to excellent validity, reliability, and responsiveness to change^11^. The questionnaire comprises 15 items and is divided into the pain and function subscales. The total score is the sum of the pain and function scores, which ranges from 0 to 100. The lower score reflects less pain and less difficulty on daily activities.

### Disabilities of arm, shoulder, and hand (DASH)

The DASH score is obtained from a self-reported questionnaire which reflects disabilities or difficulties in using upper extremity during usual daily activities and specific activities^12^. It comprises 3 modules: the disability/symptom module, working module, and sports/performing arts module. The participants in this study were asked to answer the questionnaire only in the first module about disabilities or symptoms during usual daily activities. The score ranges from 0 to 100. The lower the score the lower difficulties or symptoms during usual activities.

### Wrist range of motion

The wrist range of motion was measured for flexion-extension and pronation-supination motion of the wrist by using a goniometer. Each range of motion is the average of two assessments at each time point.

### Grip strength

Grip strength was measured using a Jamar Hydraulic Hand Dynamometer. The participants were asked to sit upright holding the dynamometer with the tested hand. The elbow was flexed to 90 degrees in neutral wrist position. From this position, participants were asked to test their grip strength three times for each hand. The grip strength of each hand was calculated by averaging the three results. The contralateral grip strength was tested for an individual reference.

### Visual analog scale (VAS) pain score

Participants were asked to rate their pain at rest and pain with activity for the past week using the VAS, which ranges from 0 to 10. A higher score reflects a higher pain level of the participants.

### Radiographic parameters

The standard true anteroposterior view and the true lateral view of the wrist radiograph were performed to measure radiographic parameters. Ulnar variance, radial inclination, and radial tilt were measured twice for each parameter at different times and the average of the two measurements was calculated. Radiographs were obtained at the immediate postoperative period and 2 weeks, 6 weeks, and 3 months after the operation. The immediate postoperative radiograph was used as a reference for the evaluation of fracture displacement at different timepoints.

### Statistical analysis

The minimum clinically important difference of the PRWE score was 11.5 points^13^. This study aimed to identify PRWE change during the follow-up period of two independent DRF patient groups. Twenty-two participants per group were required to detect the effect size of the difference with an 80% statistical power with a significance level of 0.05. With an expected 5% drop-out rate, a total of 48 participants (24 participants per group) were required for this study.

Baseline characteristics were compared between groups using the Chi-square test or Fisher’s exact test for categorical data. Continuous variables were compared using an independent t-test for normal distribution data. Linear mixed models for repeated measures were used to detect differences in outcomes between groups with the effect of time. Between-group differences and within-group differences were investigated for all continuous outcome measures. The p-values of < 0.05 were considered statistically significant. The analysis of this study was conducted based on intention-to-treat analysis. All statistical analyses were performed using IBM SPSS Statistics for Windows, Version 23.0 (IBM Corp., Armonk, NY, USA).

### Ethics approval

The ethical approval for this study was obtained from Siriraj Institutional Review Board (Certificate of Approval no. Si 434/2017).

### Informed consent

Written informed consent was obtained from all participants included in the study.

### Consent to participate

Written informed consent was obtained from all subjects before the study.

### Consent for publication

The authors would like to declare that the study is original research that has not been published elsewhere and is not under consideration by another journal. All the authors have approved the enclosed manuscript.

## Results

**Figure 1:**
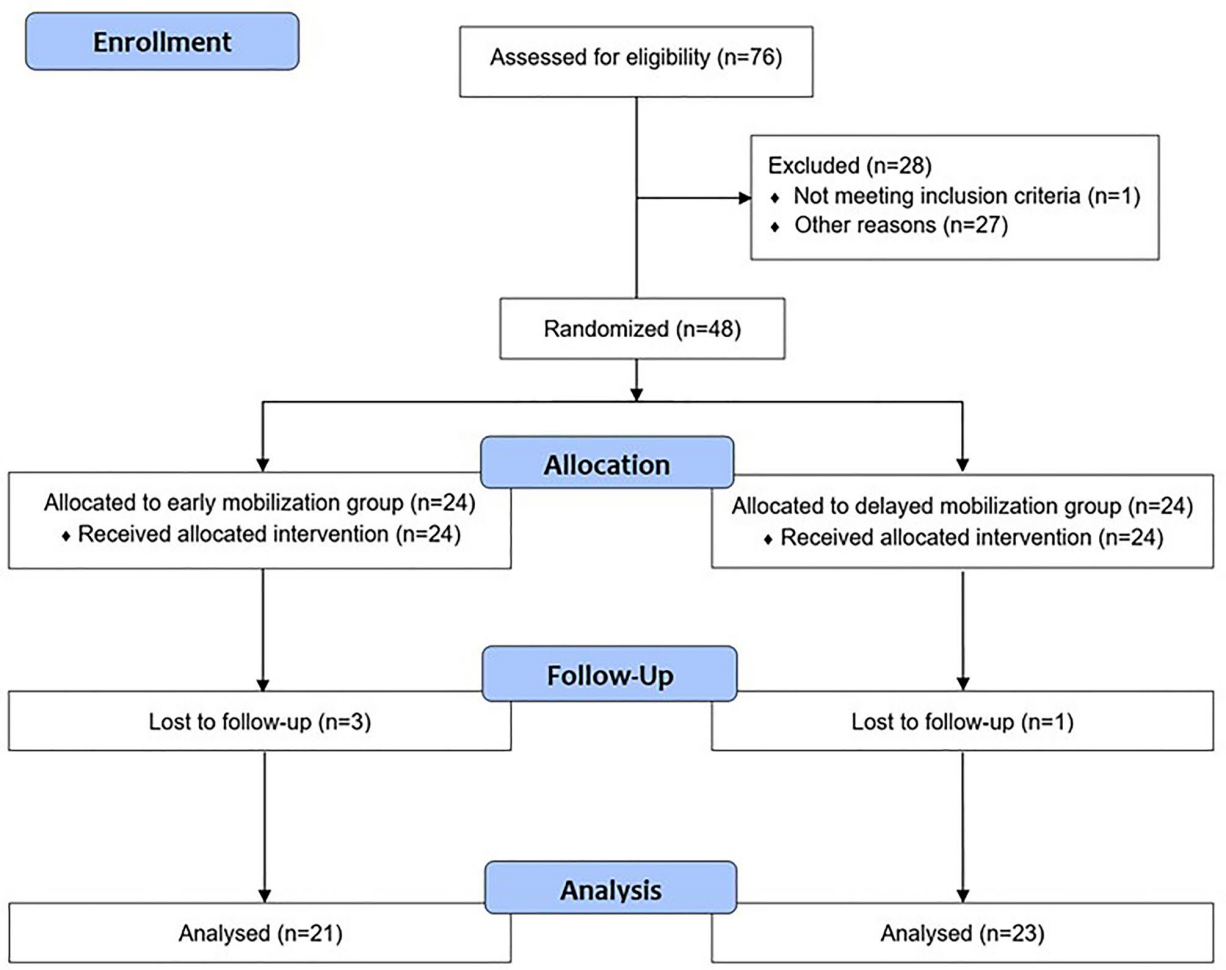
Consolidated standards of reporting trials (CONSORT) flow diagram.

The CONSORT flow diagram is presented in Fig. 1. A total of 76 patients were screened for eligibility criteria. However, twenty-eight patients were excluded from the study. One patient had a time to surgery of more than 4 weeks due to late presentation. One patient had impaired cognitive function and could not comply with postoperative care protocols and outcome measurements. Ten patients with multiple traumas and one patient with open fracture of the distal radius were excluded from the study. Four patients had a history of previous wrist fracture. We excluded 11 patients who had concomitant ulnar styloid fracture or TFCC injury needing fixation or repair. Further postoperative immobilization were needed in those patients. Dynamic fluoroscopic examination of the wrist was performed by full passive wrist motions in both frontal and sagittal planes. However, no patient was excluded from the study due to unstable fracture after volar fixed-angle plate fixation as assessed by intra-operative fluoroscopy. Therefore, forty-eight participants were consecutively enrolled into the study and randomized into 2 groups (24 participants for each group). However, three participants in the early mobilization group and 1 participant in the delayed mobilization group were lost to follow-up at the end of the study. All participants received the assigned intervention throughout the study.

Most participants were female with mean age of 54.4 years in the early group and 56.2 years in the delayed group. Most fractures occurred from a simple fall and injured the nondominant hand (58%). Fracture severity was classified by AO/OTA classification, which showed that most injuries were complete intraarticular fractures (type C) in both groups. The mean time to surgery in the early group and the delayed group was 11 and 12 days after the injury, respectively. The demographic data of participants are presented in Table 1.

**Table 1:** Demographic data.

Demographic data	Total (n = 48)	
	Early mobilization (n = 24)	Delayed mobilization (n = 24)
Age* (years)	54.4 (14.4)	56.2 (13.9)
Female gender	15 (63%)	16 (67%)
Injury to the nondominant hand	14 (58%)	14 (58%)
**AO/OTA classification**		
2R3A2	1 (4.2%)	0
2R3A3	4 (16.7%)	2 (8.3%)
2R3B2	4 (16.7%)	1 (4.2%)
2R3B3	3 (12.5%)	2 (8.3%)
2R3C1	4 (16.7%)	11 (45.8%)
2R3C2	6 (25%)	5 (20.8%)
2R3C3	2 (8.3%)	3 (12.5%)
Time to surgery* (days)	11 (11)	12 (11)

*Data were presented as median (interquartile range).

**Figure 2:**
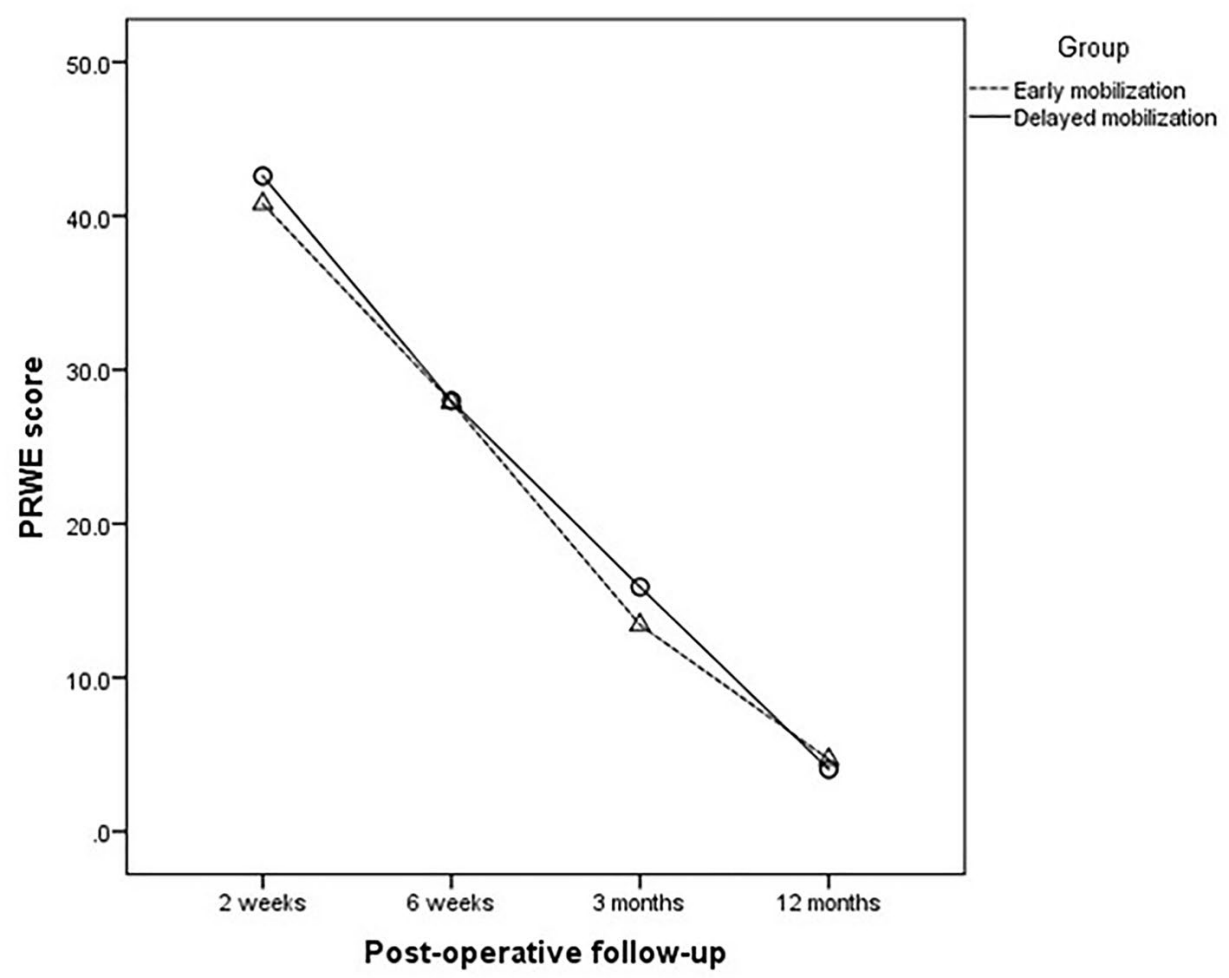
Changes of postoperative patient-rated wrist evaluation (PRWE) scores between the early and delayed mobilization groups.

The PRWE scores of both groups significantly improved during the follow-up period. The primary outcome of this study was the mean PRWE score at 3 months after the surgery which showed no statistically significant difference between the two groups (p = 0.591). The mean PRWE score at 3 months was 13.4 (SD 9.8, 95% confidence interval (CI) 8.9–17.9) in the early group and 15.3 (SD 12.6, 95% CI 9.8–20.7) in the delayed group. The PRWE scores at 2 weeks, 6 weeks, and 12 months showed similar results to those of the primary outcome. There were no statistically significant differences between groups at each follow-up (Fig. 2).

The functional outcomes of the two groups are shown in Table 2. The DASH scores at 2 weeks, 6 weeks, and 3 months did not show statistically significances between the two groups. Scores improved significantly over the follow-up period. The mean pain score during activity in the delayed mobilization group was slightly greater than that of the early mobilization group (mean 3.2 and SD 1.8 in delayed group, mean 2.8 and SD 1.8 in early group). However, there was no statistically significant difference between groups (p = 0.463). The pain scores at rest and during activity did not show statistically significant differences between the early and delayed mobilization groups at each follow-up period. The early mobilization group showed better grip strength at the 2-week follow-up but did not reach a statistically significant difference (p = 0.323). There were also no statistically significant differences at the 6-week and 3-month follow-ups. Patients in both groups could regain their grip strength to more than 50% of that of the contralateral side at 3 months.

**Table 2:** Functional outcomes between the two groups at each follow-up period.

Functional outcomes	Early mobilization (n = 24)		Delayed mobilization (n = 24)		p-value
	Mean (SD)	95% CI	Mean (SD)	95% CI	
**PRWE**					
2 weeks	40.5 (16.3)	33.5–47.6	43.4 (17.8)	35.9–50.9	0.564
6 weeks	27.5 (12.7)	21.9–33.2	27 (18.7)	18.9–35.1	0.904
3 months	13.4 (9.8)	8.9–17.9	15.3 (12.6)	9.8–20.7	0.591
12 months	4.6 (2.9)	3.3–5.8	4 (2.6)	2.9–5.1	0.504
**DASH**					
2 weeks	46.4 (19.7)	37.9–54.9	50.5 (20.7)	41.7–59.2	0.493
6 weeks	28.8 (12.5)	23.2–34.3	27.8 (15.4)	21.2–34.5	0.821
3 months	16.7 (14.7)	10–23.4	16 (14.6)	9.7–22.3	0.876
**VAS at rest**					
2 weeks	0.8 (1)	0.3–1.2	0.8 (1.1)	0.3–1.2	0.942
6 weeks	0.3 (0.9)	0–0.7	0.2 (0.5)	0–0.4	0.658
3 months	0.1 (0.4)	0–0.3	0 (0.2)	0–0.1	0.613
**VAS with activity**					
2 weeks	2.8 (1.8)	2–3.6	3.2 (1.8)	2.4–3.9	0.463
6 weeks	2 (1.5)	1.3–2.6	1.5 (1.2)	1–2	0.244
3 months	0.8 (1.1)	0.3–1.3	0.4 (0.8)	0.1–0.8	0.204
**Grip strength of the affected side (%)**					
2 weeks	25.3 (17.8)	16.5–34.1	19.5 (16.6)	11.3–27.8	0.323
6 weeks	41 (15.9)	32.9–49.2	44.8 (21.3)	34.3–55.4	0.554
3 months	56.1 (18.3)	45.5–66.6	55.9 (27)	42.8–68.9	0.982
**Flexion–extension arc of motion (degree)**					
2 weeks	54.1 (22.7)	44.3–64	45.3 (21.4)	36.1–54.6	0.184
6 weeks	84 (29.7)	71.2–96.8	78.8 (24.2)	68.3–89.2	0.517
3 months	100.6 (25.1)	88.8–112.4	103.6 (27)	92–115.3	0.708
**Pronation-supination arc of motion (degree)**					
2 weeks	106.7 (35.8)	91.3–122.2	101.3 (46.9)	81–121.6	0.661
6 weeks	142.2 (35.2)	127–157.4	143.3 (35.3)	128–158.5	0.917
3 months	160.8 (16.6)	153–168.5	165.2 (20.9)	156.2–174.2	0.446

Independent t-test. *PRWE* patient-rated wrist evaluation, *DASH* disabilities of arm, shoulder, and hand, *VAS* visual analog scale pain score.

The flexion-extension arc of motion was slightly better in the early mobilization group at 2 weeks and 6 weeks after the surgery. The differences did not show statistical significance at any timepoint. The pronation-supination arc of motion was better in the early mobilization group only at 2 weeks but there were no statistically significant differences between groups. Both groups could regain nearly normal wrist motion at 3 months.

There were no statistically significant differences in radial inclination, ulnar positive variance, and radial tilt between the early and delayed mobilization groups at any timepoint compared to the immediate postoperative radiograph (Table 3).

**Table 3:** Radiographic outcomes.

Changes of radiographic parameters	Early mobilization (n = 24)		Delayed mobilization (n = 24)		p-value
	Mean (SD)	95% CI	Mean (SD)	95% CI	
**Radial inclination (degree)**					
2 weeks—post operation	0.4 (2.9)	− 0.8–1.7	1.3 (2.6)	0.2–2.4	0.283
6 weeks—post operation	0.7 (2.9)	− 0.5–2.0	0.7 (2.8)	− 0.6–1.9	0.918
3 months—post operation	1.0 (3.1)	− 0.4–2.4	0.8 (3.3)	− 0.6–2.2	0.824
**Ulnar positive variance (mm)**					
2 weeks—post operation	− 0.1 (0.5)	− 0.4–0.1	− 0.4 (0.5)	− 0.6– − 0.1	0.160
6 weeks—post operation	− 0.4 (0.6)	− 0.7– − 0.2	− 0.5 (0.7)	− 0.8– − 0.2	0.675
3 months—post operation	− 0.4 (0.6)	− 0.7– − 0.2	− 0.6 (0.6)	− 0.9– − 0.4	0.319
**Radial tilt (degree)**					
2 weeks—post operation	− 0.1 (2.0)	− 1.0–0.8	0.5 (3.6)	− 1.1–2.0	0.531
6 weeks—post operation	0.7 (2.8)	− 0.5–1.9	− 0.3 (3.3)	− 1.8–1.1	0.250
3 months—post operation	1.0 (2.7)	− 0.3–2.2	0 (3.4)	− 1.5–1.5	0.311

Independent t-test.

Table 4 demonstrated the time effect, between-group effect, and interaction effect of functional outcomes and radiographic parameters by using a linear mixed model. All functional outcomes showed an improvement over time (p < 0.001). However, functional outcomes did not show statistically significant differences between groups and no interaction was found between the time and treatment groups. No statistically significant differences in radiographic parameters were found between groups. There was a slight decrease ulnar positive variance over time in both groups which showed statistically significant differences (F test = 7.21, p-value = 0.001). However, the changes of ulnar variance were not clinically meaningful.

**Table 4:** Between-group differences of the functional outcomes and radiographic parameters.

Functional outcomes	Time effect		Between-group effect		Interaction effect	
	F test	p-value	F test	p-value	F test	p-value
PRWE	214.12	< 0.001*	0.115	0.736	0.085	0.772
DASH	74.74	< 0.001*	0.017	0.897	0.352	0.705
VAS at rest	18	< 0.001*	0	1	0.008	0.929
VAS with activity	70.77	< 0.001*	0.105	0.747	1.99	0.166
Grip strength	94.44	< 0.001*	0.128	0.723	0.79	0.381
Flexion–extension arc of motion	121.8	< 0.001*	0.122	0.728	0.829	0.44
Pronation-supination arc of motion	97.69	< 0.001*	0.006	0.941	0.269	0.607
Change of radial inclination	0.221	0.802	0.006	0.936	1.13	0.328
Change of ulnar variance	7.21	0.001*	1.057	0.31	0.345	0.709
Change of radial tilt	1.34	0.269	0.56	0.458	1.14	0.326

Linear mixed model. *PRWE* patient-rated wrist evaluation, *DASH* disabilities of arm, shoulder, and hand, *VAS* visual analog scale pain score.

Four participants developed carpal tunnel syndrome during the study period (2 participants in each group). One participant in each group had carpal tunnel syndrome after the injury and underwent simultaneous carpal tunnel decompression during the open reduction and internal fixation with volar fixed-angle plate of the distal radius operation. Only one participant in the delayed mobilization group developed carpal tunnel syndrome at 3 months after the surgery and underwent a carpal tunnel decompression procedure. No participants required plate removal during the 12-month study period.

## Discussion

Some previous studies have demonstrated the functional outcomes of various postoperative protocols, with varied outcomes depending on their postoperative immobilization protocols. An increase in the duration of the immobilization period might affect functional outcomes in the early postoperative period. However, the longer-term follow-up showed comparable results. A prospective randomized controlled study by Brehmer et al*.* suggested that the accelerated rehabilitation facilitated an earlier return to function compared to that of the standard protocol. Patients who started wrist motion exercise immediately after volar fixed-angle plating of DRF and strengthening exercise at 2 weeks (accelerated rehabilitation protocol) regained a better mobility, strength, and functional outcomes than those following the standard protocol, which started with a passive range of motion and strengthening exercise at 6 weeks postoperation^14^.

Quadlbauer et al*.* compared two different rehabilitation protocols after volar distal radius plate fixation between early rehabilitation with a removable thermoplastic splint for 1 week and immobilization with a nonremovable plaster cast for 5 weeks^15^. The results showed that the early mobilization group had a significantly better range of motion, grip strength, PRWE score, and DASH score at the 6-week follow-up, which was only 1 week after the cast was removed in the immobilization group. The grip strength and range of motion were also better in the early mobilization group at the 9-week follow-up, but the self-reported functional scores were not significantly different between groups. Andrade-Silva et al*.* performed a randomized controlled trial between early mobilization without external splint and 2-week short arm splint immobilization after volar distal radius plate fixation and showed a slight increase with tramadol usage in the early mobilization group during hospital stay^16^. However, no statistical significance was found between groups. The pain score and functional scores were similar between the two groups. Zeckey et al*.* performed a randomized controlled study comparing early mobilization and 4-week immobilization with commercial wrist orthoses after DRF fixation with volar fixed-angle plate^17^. The modified Mayo wrist score was significantly better in the early mobilization group at 6 weeks but there were no differences between groups at any further clinical courses. As per the above-mentioned studies, prolonged immobilization seem to worsen the postoperative functional outcomes compared to the early mobilization protocol. The longer duration of wrist immobilization would delay recovery of wrist motion, grip strength, and patient daily activities.

Our study results revealed no difference in functional outcomes between the early mobilization group and the 2-week delayed mobilization group after DRF fixation with volar fixed-angle plate over the 12 months follow-up period. In addition, radiographic parameters and complications did not differ between groups. These findings were in line with those of a retrospective review by Duprat et al*.* comparing early mobilization and 2-week immobilization with short arm splint in patients who underwent distal radius plate fixation^18^. The study results showed no statistically significant differences in terms of range of motion, pain score, QuickDASH score, PRWE score, and grip strength between groups. However, the radiographic evaluation determining losses of reduction did not reported in the study.

Despite the clear benefits of early rehabilitation demonstrated in various studies, these findings have not altered the practice of hand surgeons, as reflected by a survey of fellowship-trained hand surgeons. Only 3.9% of surgeons did not perform wrist immobilization after the operative fixation of DRF, and only 8.1% of the surgeons immediately initiated postoperative range-of-motion exercises^19^. Most surgeons preferred postoperative immobilization and postponed the time to initiate range-of-motion exercises due to doubtfulness regarding fixation stability, which might lead to fracture displacement over time.

The evidence from our study convincingly confirmed the benefit of the biomechanical properties of fixed-angle plate fixation in DRF. Early mobilization without additional postoperative external immobilization did not increase pain, risk of fracture displacement, or compromised functional outcomes.

There were some limitations to this study. We enrolled only patients with acute DRF, injured within 4 weeks before the operation. The study excluded patients with instability of the distal radio-ulnar joint requiring additional fixation or repair and unstable fractures after volar-fixed angle plate fixation which require additional external immobilization. Therefore, we could not conclude the similar results to these excluded conditions. The VAS pain scores did not differ in this study at any follow-up period. However, the quantity of postoperative analgesic medications and compliance were not recorded and there might be differences in analgesic drug use or patient satisfaction between groups. Further studies should focus on these issues.

## Conclusions

The early mobilization protocol immediately after operation is safe and shows functional results comparable to those of the delayed mobilization protocol. Additional external immobilization is not necessary after open reduction and internal fixation with volar fixed-angle plate of DRF.

## Data Availability

The datasets used or analyzed during the study are available from the corresponding author on reasonable request.
